# Editorial

**DOI:** 10.1080/17571472.2015.1082342

**Published:** 2015-09-28

**Authors:** Paul Thomas

This Issue coincides with two important developments for LJPC:

1. Our new Publishing House – Taylor and Francis.

Thank you to all our friends at Radcliffe who have nurtured us from stumbling beginnings to present strength that bears testament to great collaborative working. Welcome to our new friends at Taylor and Francis – we look forward to developing a strong relationship with you, and take advantage of your extensive reach.

2. Integrated care becoming the new NHS Watchword.

It seems that every NHS document now identifies integrated care as a central policy. This is very encouraging for those of us who have been advocating this for so long. We welcome new readers, and new authors, to advance understanding of how to make holistic community-oriented integrated care a reality.

In this Issue, you can read about the ideas that emerged from the (London) City Health Conference at which academics and practitioners discussed practical ways to create community-oriented integrated care. Majeed’s article provides a cautionary note about the financial squeeze on primary care in the UK – if you want to create community-oriented integrated care you have to resource it.

This Issues includes another wonderful example of learning and change that can happen when specialists and generalists share care and share learning. Mills and Devendra present a case study of steroid-induced diabetes for which the HbA1C is a useless measure.

This issue includes a debate about the ethical dilemmas facing GPs and other healthcare professionals when giving sexual health advice and contraception to young women. Bow critiques a previous LJPC paper by Papanikitas. Papanikitas and Spicer respond. What do you think? How often is this an ethical problem for primary care clinicians?

Oesterling et al. provide a good example of how general practice can evaluate interventions for patient care. They explain how the ankle-brachial pressure index is a simple but helpful way to help understand atypical leg pain.

Paul Thomas paul.thomas7@nhs.net

